# Call to Action for Dengue Vaccine Failure

**DOI:** 10.3201/eid1908.121864

**Published:** 2013-08

**Authors:** Suresh Mahalingam, Belinda L. Herring, Scott B. Halstead

**Affiliations:** Griffith University, Southport, Queensland, Australia (S. Mahalingam, B.L. Herring);; Dengue Vaccine Initiative, Rockville, Maryland, USA (S.B. Halstead)

**Keywords:** dengue virus, DENV, dengue, viruses, vaccine, vaccine failure, antibody-dependent enhancement of infection

**To the Editor:** Dengue is one of the most widespread infectious diseases globally; transmission now occurs in 128 countries. Although dengue virus (DENV) control strategies have targeted vector control and disease surveillance, the development of an effective vaccine is the holy grail of prevention.

Dengue vaccine development has spanned many decades. A candidate vaccine (Sanofi Pasteur, Swiftwater, PA, USA) containing all 4 DENV serotypes is in advanced clinical testing. However, when given to school children in Thailand, this live-attenuated, tetravalent, dengue–yellow fever 17D chimeric virus vaccine showed major but incomplete efficacy against 3 of the 4 DENV serotypes (DENV 1 [61.2%], DENV-3 [81.3%], and DENV-4 [89.9%]) in the intention-to-treat group but no protection against DENV 2, the most pathogenic of the DENV serotypes (*1*).

Two observations from the efficacy trial in Thailand provide insights into protective immunity that could greatly improve second-generation vaccines. The first observation was that a single dose of 4 live-attenuated chimeric DENVs given subcutaneously at a single site failed to raise type-specific protective immunity against the 4 DENV serotypes, and 2) doses 2 and 3 of the Sanofi Pasteur vaccine given to children over a 1-year period failed to improve efficacy outcomes. These results were obtained even though 91% of the children had circulating dengue or Japanese encephalitis antibodies before vaccination, neutralizing antibodies developed to all 4 DENVs, and neutralizing antibody titers increased 2–3 fold after 3 doses of vaccine in 80%–90% of vaccinated children.

The inability of a mixture of 4 dengue chimeric viruses to elicit an initial primary neutralizing antibody response in nonhuman primates and susceptible humans was recognized during preclinical testing and explained by the phenomenon of interference (*2*). Although protective immunity was raised in susceptible rhesus monkeys inoculated with all 4 DENVs at a single site, inoculation of 4 chimeric dengue viruses at 1 or 2 sites did not result in neutralizing antibody responses to all 4 DENV (*3*). Studies on human primary immune responses to dengue infection have identified critical attachment sites on the virion for neutralizing antibodies (*4*). Serum samples from children given ≥1 doses of the dengue chimeric vaccine can now be tested for primary neutralizing antibody responses to each of the 4 DENVs. As an alternative, antibody-secreting cells may be isolated and their products identified by using methods as described for dengue-infected children in Nicaragua (*5*).

Infections with 2 different DENVs can protect against severe disease during subsequent infections (*6*). It has therefore been assumed that persons with DENV neutralizing antibodies are protected against infection. In clinical testing of the Sanofi Pasteur vaccine, failure of multiple booster doses to show protection was unexpected because the children were already substantially immune from prior exposure to DENV or Japanese encephalitis vaccine. When the tetravalent dengue chimeric vaccine was given to partially dengue–immune children and adults in the Philippines, a broad neutralizing antibody response was observed after administration of only 2 vaccine doses (*7*).

We believe that the unanticipated results of the dengue vaccine efficacy trial in Thailand call for new methods of assessing dengue immunity. Myeloid cells are major targets of dengue infection in humans. We and others have described the unique biologic responses when dengue virus–antibody complexes are presented to myeloid cells (*8*). There is evidence that DENV neutralization titers differ when the same antibodies are assayed in epithelial and Fc-receptor–bearing cells (*8*). Recent work suggests that primary monocytes and macrophages may not respond in exactly the same fashion to infection by DENV immune complexes (*8*). Few relevant studies exist in the literature, and most focused on DENV-2. Detailed studies on innate immune responses in human myeloid cells with a variety of dengue immune complexes should proceed forthwith.

To our knowledge, only once has an in vitro test correctly predicted which children would be susceptible or have silent infections accompanying a second heterotypic dengue infection (*9*). This was determined by using serum samples collected before a second dengue infection and testing these serum samples at low dilutions for their ability to protect primary human monocytes from DENV-2 infection or antibody-dependent enhanced infection. During development of the Sanofi Pasteur tetravalent chimeric dengue vaccine, serum samples from vaccinated persons were routinely tested for neutralization of DENV in an epithelial cell line (*10*). In addition to assaying for antibodies directed at the quaternary site described by de Alwis et al. (*4*), we suggest that serum samples from vaccinated persons be tested for neutralization of all DENVs in primary human myeloid cells.

Although human Fc-receptor cell lines may be convenient for assaying DENV antibodies, decisions regarding their use should be deferred until they are shown to model primary myeloid cells. Because antibody titers often wane after vaccination, the ability of serum samples from vaccinees to protect against infection of myeloid cells with the 4 DENVs should be studied over many years. Changes to in vitro systems for measuring immune responses after dengue vaccination may provide a better surrogate of protection by realigning antibody measurement systems to our contemporary understanding of the pathogenesis of this complex disease.
